# Successful Management of a Renin-Secreting Tumor in Pregnancy: A Case Report

**DOI:** 10.7759/cureus.73281

**Published:** 2024-11-08

**Authors:** Toshiki Tamura, Ken Maekawa, Kentaro Ishida, Hikaru Kiyokawa, Hiroyuki Ohnishi, Takafumi Nonogaki

**Affiliations:** 1 Obstetrics and Gynecology, Osaka Red Cross Hospital, Osaka, JPN; 2 Obstetrics and Gynecology, Saiseikai Noe Hospital, Osaka, JPN; 3 Urology, Osaka Red Cross Hospital, Osaka, JPN; 4 Gynecology and Obstetrics, Kyoto University Graduate School of Medicine, Kyoto, JPN; 5 Obstetrics and Gynecology, Kurashiki Central Hospital, Okayama, JPN

**Keywords:** hypertension, pregnancy-induced hypertension, renal, renin, tumor

## Abstract

Hypertension that develops during early pregnancy has several causes. However, there have been no reported cases of full-term delivery, particularly when it is caused by renin-secreting renal tumors. We present the case of a 28-year-old pregnant patient who delivered with a renin-secreting renal tumor and provide a literature search of similar cases. A patient with no history of hypertension was referred to our hospital for severe hypertension during early pregnancy and was admitted for antihypertensive and aspirin therapy. The renin level of the patient was abnormally high, and a renal tumor was detected in the right kidney. Robot-assisted partial nephrectomy was performed at 16 weeks of gestation, after which antihypertensive medication was discontinued because hypertension was not observed. The pregnancy progressed favorably, leading to an uncomplicated delivery of a healthy baby at 40 weeks of gestation. The successful treatment of our patient can be attributed to three key factors: early hospitalization for strict blood pressure control, aspirin administration, and prompt diagnosis and treatment. Medication and thorough investigation of secondary hypertension are crucial in pregnant women with hypertension.

## Introduction

According to the National Center for Health Statistics in the United States, the prevalence of hypertension during pregnancy has exhibited a consistent upward trend over the past five decades, attaining a rate of 55.9% among women who gave birth in 2019 [1]. Hypertension during pregnancy can lead to severe maternal complications, including cerebrovascular events, eclampsia, organ dysfunction, and mortality. For the fetus, potential consequences include intrauterine growth restriction, low birth weight, preterm delivery, placental abruption, stillbirth, and neonatal mortality. Pregnancy-induced hypertension has various causes, with approximately 10% classified as secondary hypertension. Although rare, there have been reports of secondary hypertension resulting from renal tumors. This type of secondary hypertension occurs either through activation of the renin-angiotensin-aldosterone system (RAAS) due to the proliferation of juxtaglomerular cells in the kidney or through renal artery narrowing resulting from tumor growth. RAAS activation can cause severe hypertension and hypokalemia, which may lead to headaches, dizziness, muscle weakness, edema, and arrhythmias. Diagnosis typically relies on elevated blood renin levels and imaging studies. Generally, RAAS inhibitors, such as angiotensin-converting enzyme (ACE) inhibitors and angiotensin II receptor blockers (ARBs), are used to treat hypertension. However, administering these medications during pregnancy can harm fetal kidneys, leading to oligohydramnios, pulmonary hypoplasia, limb contractures, hypotension, and, in many cases, fetal or neonatal death. Therefore, surgical resection is often the preferred approach. These factors make managing renin-producing renal tumors during pregnancy exceptionally challenging. The exact prevalence of angiomyolipoma is unknown, although it is more common in women than in men. Rapid tumor growth during pregnancy is frequently reported, with tumor rupture being the most common complication [2,3]. However, renin production has not been reported since angiomyolipomas are benign tumors composed of smooth muscle, adipose tissue, and vascular endothelial cells.

In 2001, Henderson et al. reported the first case of a renin-secreting kidney tumor during pregnancy [4]. Although several such cases have been reported thereafter [5-11], uncomplicated term delivery did not occur in these cases, owing to delayed diagnosis or inadequate antihypertensive therapy. To the best of our knowledge, this case report describes the first full-term healthy live birth in a pregnancy complicated by a renin-secreting renal tumor, the renin-secreting mechanism of renal tumors during pregnancy, and early diagnosis and treatment considerations.

## Case presentation

A 28-year-old primigravid patient, who had not been previously diagnosed with hypertension, visited a local physician with a chief complaint of amenorrhea and pregnancy. Family history revealed hypertension in the father, mother, and grandmother. The blood pressure of the patient was 130/90 mmHg and 174/122 mmHg at eight and 12 weeks of gestation, respectively. The patient was referred to our department with severe chronic hypertension. The patient’s blood pressure was 151/88 mmHg on the first visit to our hospital at 12 weeks of gestation, and the patient was immediately admitted for antihypertensive therapy and close monitoring. Endocrinological studies, abdominal ultrasonography, abdominal magnetic resonance imaging (MRI), and renal vascular resistance tests were performed. The patient was treated with aspirin and antihypertensive medication. Treatment was initiated with monotherapy of nifedipine 40 mg/day. Blood pressure control was insufficient, so nifedipine was increased to 80 mg/day, and methyldopa 375 mg/day was concurrently added. Furthermore, labetalol 150 mg/day was added, and, subsequently, methyldopa was increased to 750 mg/day. Following this, labetalol was increased to 450 mg/day. As a final step, benidipine 4 mg/day was introduced and then increased to 8 mg/day. The doses mentioned at each stage represent the total daily oral intake.

**Figure 1:**
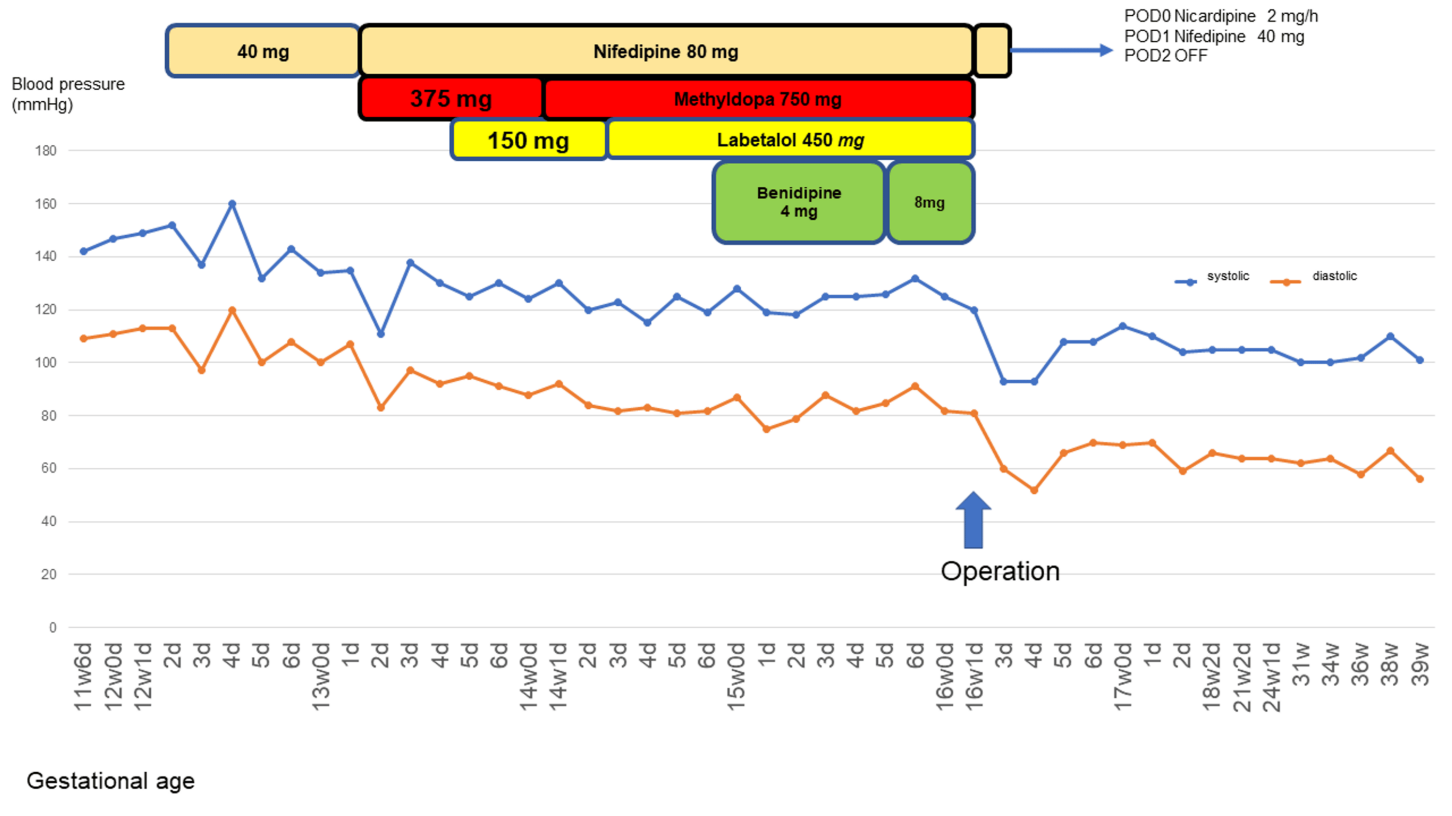
Antihypertensive drug and blood pressure trends Blood pressure was resistant to treatment but decreased rapidly after surgery.

**Figure 2:**
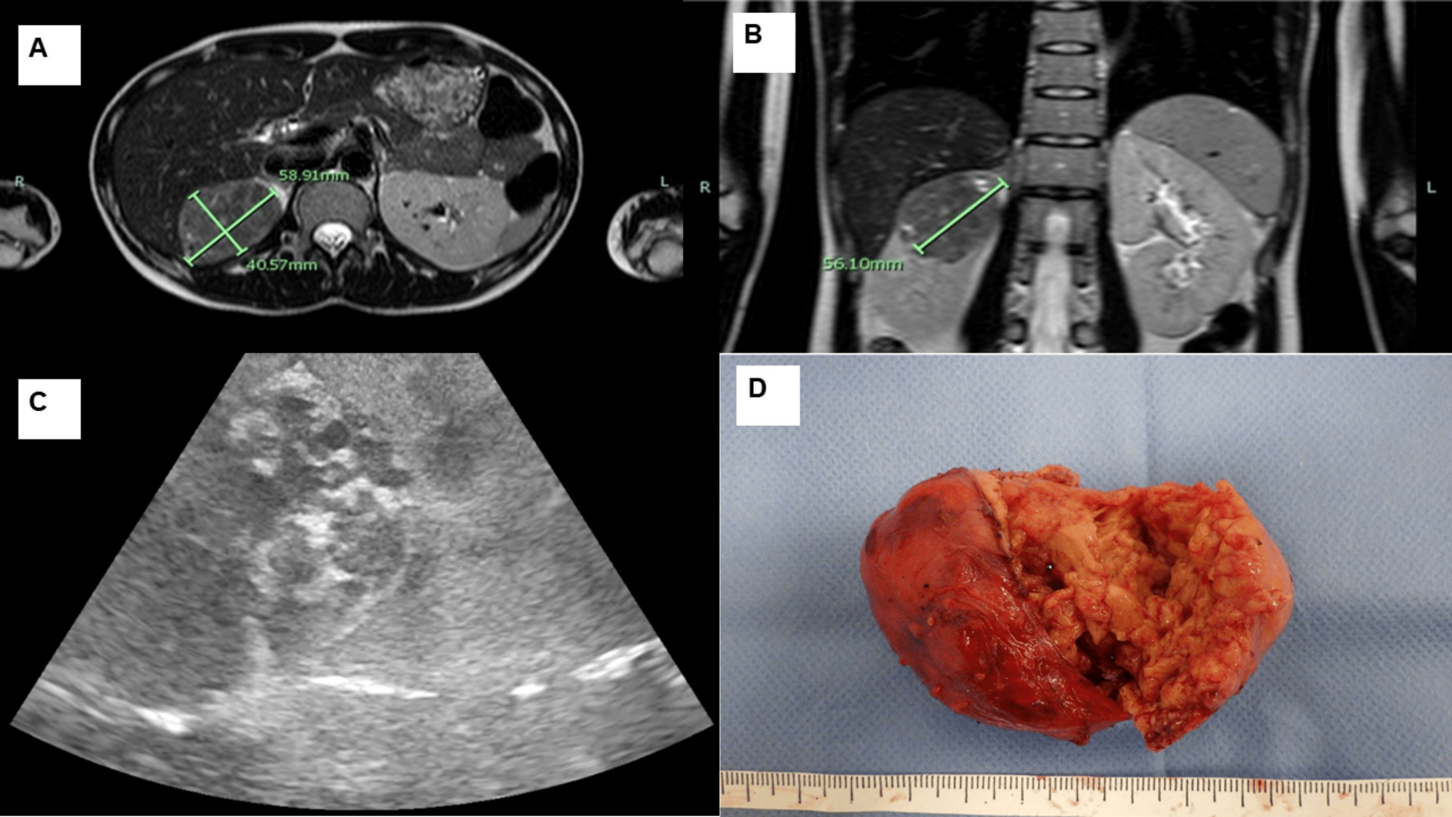
Magnetic resonance imaging (MRI), intraoperative echography, and gross image of the tumor Magnetic resonance imaging showing a 5.8 × 4.0 × 5.6 cm tumor originating from the upper pole of the right kidney. A: T2-weighted imaging (WI), transverse section; B: T2WI, coronal section; C: intraoperative echogram showing a well-defined borderline mass; D: Resected right kidney tumor with the size of 41 x 58 x 49 mm.

Antihypertensive drugs and blood pressure trends are shown in Figure 1. Endocrinological examination revealed hypokalemia (serum potassium level: 2.9 mEq/L; normal range: 3.5-5.0 mEq/L) and abnormally high renin levels (114 ng/mL · h). Abdominal ultrasound showed a 5.4 × 5.3 × 4.7 cm mass with well-defined borders and a heterogeneous internal calcification at the upper pole of the right kidney. Furthermore, there was no apparent difference in flow velocity at the origin of the right renal artery compared to the left, and no evidence of renal artery stenosis (peak systolic velocities (PSV) of the right and left renal arteries were 85.8 cm/s and 60.4 cm/s, respectively; a PSV of >180 cm/s is diagnostic of renal artery stenosis) [9]. Furthermore, there was no decrease in uterine arterial vascular resistance. MRI confirmed a right renal tumor measuring 5.8 × 4.0 × 5.6 cm (Figures 2A, 2B). No bilateral enlargement of the adrenal glands was observed. We hypothesized that renin secretion by the tumor was the cause of hypertension. Following consultation with the urology department, robot-assisted partial nephrectomy was performed at 16 weeks of gestation. The intraoperative echogram is shown in Figure 2C. The surgery lasted for 3 h 52 min, with a blood loss of 50 mL. The patient was admitted to the intensive care unit after surgery. The dosage of antihypertensive medications was gradually reduced immediately after surgery and completely discontinued by the second postoperative day. Renin levels returned to normal within a week after surgery. The resected right renal tumor measured 41 × 58 × 49 mm and macroscopically appeared as a borderline, well-defined neoplastic lesion (Figure 2D).

**Figure 3:**
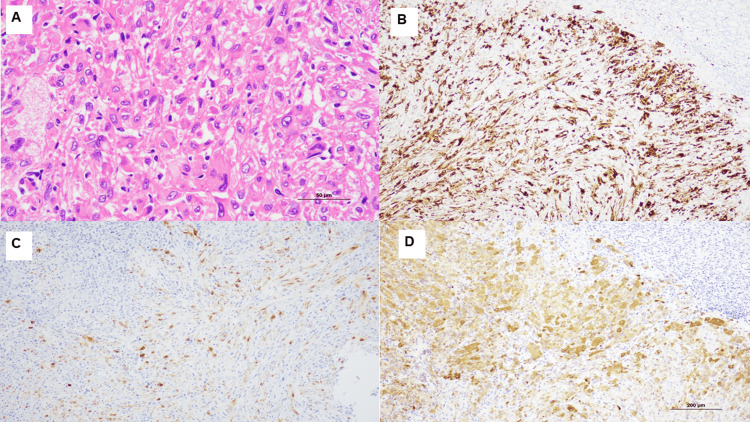
Histological and immunohistochemical analyses The tumor is composed of epithelioid and spindle cells with eosinophilic cytoplasm. The nuclei of most tumor cells are round to oval, although long spindles or bean-like shapes are intermingled (A, 40× magnification); immunostaining for renin (B, 10× magnification); Human melanin black-45 (HMB45) (C, 10× magnification); and melan A (D, 10× magnification).

On pathological examination, hematoxylin and eosin staining showed sheet-like proliferation of spindle-shaped cells with acidophilic fine granular cytoplasm, adipocytes, and microvessels, consistent with angiomyolipoma (AML) (Figure 3A). Immunostaining revealed strong anti-renin antibody staining (Figure 3B), an unusual finding in AML that suggests renin production by the tumor. Other staining results were as follows: positive for human melanin black (HMB)-45 (Figure 3C) and melan-A (Figure 3D). These melanocytic markers are typically expressed in AML and are crucial for its definitive diagnosis, distinguishing it from other renal tumors.

The patient was discharged eight days after surgery without any recurrence of hypertension. The ratio of soluble fms-like tyrosine kinase-1 to placental growth factor was normal at 14, 16, 18, and 24 weeks of gestation. The pregnancy continued uneventfully, and a healthy baby weighing 3,232 g was born via spontaneous vaginal delivery with no postpartum complications. The placenta weighed 650 g, and placental pathology showed a mild infarct but no other abnormalities. Blood pressure remained stable one month postpartum. No renal blood flow changes suggestive of renal artery stenosis were present during pregnancy or postpartum (the postpartum PSVs of the right and left renal arteries were 77.9 cm/s and 85.0 cm/s, respectively).

## Discussion

To the best of our knowledge, this is the first report of a full-term uncomplicated birth in a pregnancy complicated by a renin-producing tumor. We conducted a comprehensive literature search using the PubMed and Google Scholar databases. The search keywords used were (pregnan* OR gestation*) AND (renin-producing OR renin-secreting OR reninoma OR juxtaglomerular) AND (tumor* OR neoplasm* OR cancer*) to identify relevant case reports of renin-secreting renal tumors during pregnancy. The search period was extended from 1950 to September 2024, and we included only peer-reviewed literature published in English. The search was conducted on October 1, 2024. After the initial search, we screened the titles and abstracts to select articles that met the eligibility criteria. We also checked the reference lists of the selected articles to identify any additional relevant reports. As a result of this systematic search process, we identified eight case reports [4-11] of renin-secreting renal tumors during pregnancy. However, we acknowledge the following limitations of our study: Reports published in languages other than English may not be included. Publication bias may have led to a tendency to report positive results. Gray literature (unpublished or non-peer-reviewed literature) was not included.

The first reported pregnancy complicated by a renin-producing tumor resulted in intrauterine fetal demise (IUFD) at 19 weeks of gestation, despite tumor resection at 16 weeks of gestation [4]. Since then, several studies have documented four pregnancies with renin-secreting tumors; however, all reported pregnancies have resulted in stillbirth, premature delivery, or the decision to terminate the pregnancy early, owing to uncontrolled blood pressure despite antihypertensive drugs [4-11] (Table 1). The effects of hypertension during pregnancy on both the mother and fetus are well documented. In the mother, it can lead to perinatal complications, such as preeclampsia, stroke, heart failure, pulmonary edema, acute kidney injury, and placental abruption. For the fetus, it can result in poor growth, preterm birth, and perinatal death. If hypertension is not treated for approximately 16 weeks, the period of placental formation often becomes irreversible. Therefore, managing blood pressure during this critical period in early pregnancy is crucial for achieving favorable pregnancy outcomes.

**Table 1:** Pregnancy outcomes in pregnancies complicated by renin-producing tumors Source: Refs. [4-11]

Author	Henderson et al. (4)	Kim et al. (5)	Lachvac et al. (6)	Shin et al. (7)	Ohashi et al. (8 )	Diker-Cohen et al. (9)	Xue et al. (10)	Fu et al. (11)	Current study
Year of publication	2001	2006	2011	2012	2014	2014	2017	2023	2024
Patient age	32	22	24	N/A	32	23	31	28	28
Gravida/para status	1/0	N/A	1/0	N/A	N/A	N/A	N/A	1/0	1/0
Hemoglobin level (g/dL)	13.6	N/A	N/A	N/A	N/A	N/A	N/A	N/A	14.2
Hypokalemia (mEq/L)	Yes (3.0)	N/A	None	N/A	Yes (2.8)	Yes (N/A)	N/A	Yes (2.9)	Yes (3.0)
Renin level (ng/mL/hr)	37.6	29.8	N/A	N/A	12	20	N/A	352	114
Week of gestation when starting antihypertensive treatment	6	N/A	20	5	5	Before pregnancy	33	7	12
Week of gestation in which renal tumor was diagnosed	16	Postpartum	Postpartum	N/A	Postpartum	Postpartum	Postpartum	Postpartum	12
Week of gestation in which renal tumor surgery was performed	16	N/A	Postpartum	N/A	Postpartum	Postpartum	Postpartum	Postpartum	16
Delivery outcome	Intrauterine fetal death	N/A	Intrauterine fetal death	N/A	Cesarean	Cesarean	Cesarean	Artificial abortion	Vaginal
Week of delivery	19	N/A	28	N/A	25	26	35	7	40
Antihypertensive	Methyldopa Hydralazine Nifedipine	N/A	Metopurolol Methyldopa Amlodipine	N/A	Hydralazine Methyldopa	Methyldopa Calcium antagonist	None	None	Nifedipine Methyldopa Labetalol Benidipine
Reason for delivery	Intrauterine fetal death	N/A	intrauterine fetal death	N/A	Preeclampsia	HELLP syndrome	Elevated blood pressure	Elevated blood pressure	Labor pains
Oral aspirin therapy	None	None	None	None	None	None	None	None	Yes

For antihypertensive treatment, it is common to use one of three medications, namely, nifedipine, labetalol, or methyldopa, often in combination if the initial treatment is insufficient [1]. Angiotensin-converting enzyme inhibitors and angiotensin receptor blockers are contraindicated because of their potential toxicity to the fetus. If antihypertensive treatment is not administered, the alternative treatment would be termination of pregnancy. We initiated treatment with the most commonly used oral calcium channel blocker; however, as it was not sufficiently effective, we promptly switched to combination therapy. If the blood pressure cannot be controlled with these three drugs, fetal delivery, which at her gestational age would mean induced abortion, is necessary. She strongly desired to continue the pregnancy and undergo antihypertensive treatment. We believe that the hypertension was reversible because appropriate blood pressure was maintained during the placental formation period through antihypertensive treatment combining three medications.

Previous cases have shown that outpatient blood pressure management before placental formation is often inadequate. Additionally, failure to identify renal tumors as the underlying cause has prevented timely surgical treatment during pregnancy. Consequently, these cases have resulted in stillbirths or premature births, requiring treatment for renal tumors after delivery. In contrast, we achieved a live birth by implementing strict blood pressure control through hospitalization while pursuing definitive treatment with surgery during pregnancy.

The success of our case can be attributed to three key factors: early hospitalization for strict blood pressure control, aspirin administration, and prompt diagnosis and treatment. The patient was hospitalized and managed immediately after the first referral visit at 12 weeks of gestation, and strict antihypertensive management with multiple medications was administered. Nearly normal blood pressure was achieved. In contrast, previous cases often relied on outpatient medical management, leading to delays in adequately reducing blood pressure, which may have contributed to IUFDs. Strict blood pressure control during placentation improves perinatal outcomes.

Aspirin was administered immediately after the diagnosis of hypertension; however, its use has not been previously documented. The effectiveness of aspirin in managing hypertensive disorders during pregnancy is well-established [12]. Although the patient had no history of hypertension, a sudden increase in blood pressure early in pregnancy suggested secondary hypertension. Further investigation revealed elevated blood renin levels and renal tumors. Prompt tumor resection was performed at 16 weeks of gestation following consultation with a urologist. In contrast, most tumor resections in previous studies have been performed postpartum.

Most renin-secreting tumors are juxtaglomerular cell tumors [13,14]; however, in this case, the diagnosis was AML. Renal AML is a benign kidney tumor that originates from perivascular epithelial cells, with 78% of cases occurring in women [2]. There have been reports of AML during pregnancy, with an average age of onset of 31.4 years and an average gestational age of 27.7 weeks [3]. The mechanism by which AML produces renin is unclear. In contrast, juxtaglomerular cell tumors originate from juxtaglomerular cells and cause renin secretion due to the overactivation of the renin-angiotensin-aldosterone system. The prevalence of AML is unknown but tends to affect young women (female-to-male ratio is 2:1, average age: 27 years) [15]. Pathological examination and immunostaining were used to diagnose both conditions. Renal AML shows characteristic fat cells and proliferation of small blood vessels and is positive for melan-A, HMB-45, and smooth muscle actin [16]. Juxtaglomerular cell tumors are positive for renin, vimentin, and CD34, but staining for CD117 and smooth muscle actin is inconsistent. In this case, spindle cell proliferation with eosinophilic granular cytoplasm, fat cells, and small blood vessels, characteristic of angiomyolipoma, was observed, and a diagnosis of renal angiomyolipoma was made based on immunostaining. Although the exact mechanism is unclear, the positive renin antibody indicated that renin was being produced by renal angiomyolipoma. However, a recent report by Miyauchi et al. showed that renal stromal fibroblasts produce renin [17]. This case supports these findings. It is possible that the mechanism of renin production differs between juxtaglomerular cell tumors and renal stromal fibroblasts. Juxtaglomerular cell tumors continuously produce renin, whereas Miyauchi et al. [17] suggested that renin production by renal stromal fibroblasts is triggered under anemic conditions. In our case, there was no pre-pregnancy hypertension, and the patient’s blood pressure rapidly increased after pregnancy. Unlike juxtaglomerular cell tumors, which consistently produce renin, it is hypothesized that physiological changes due to pregnancy stimulate renin production by renal stromal fibroblasts in AMLs. However, further studies are required to clarify the detailed mechanisms.

In this case, the tumor was removed via robot-assisted surgery. Several robotic renal surgeries have been safely performed during pregnancy, and the safety of robotic nephrectomy during pregnancy has been previously reported [18-20]. Consistent with other reports, blood pressure improved rapidly after the removal of the renal tumor, and antihypertensive medication was no longer necessary.

## Conclusions

To the best of our knowledge, this is the first reported case of a renin-secreting tumor during pregnancy that was successfully managed with good outcomes. This case underscores the importance of comprehensive evaluation and tailored management of hypertension in early pregnancy. For future cases where significant hypertension is encountered during early gestation, our experience suggests that maintaining normal blood pressure through strict management during the placental formation period is crucial. Additionally, administering aspirin, thoroughly investigating the cause of hypertension, and promptly providing fundamental treatment if curable secondary hypertension is identified are key components of effective management. These strategies may contribute to improving maternal and fetal outcomes in similar cases. Further research is needed to establish standardized protocols for managing rare causes of hypertension during pregnancy.

## References

[1] Tita AT, Szychowski JM, Boggess K (2022). Treatment for mild chronic hypertension during pregnancy. N Engl J Med.

[2] Flum AS, Hamoui N, Said MA (2016). Update on the diagnosis and management of renal angiomyolipoma. J Urol.

[3] Çetin C, Büyükkurt S, Demir C, Evrüke C (2015). Renal angiomyolipoma during pregnancy: case report and literature review. Turk J Obstet Gynecol.

[4] Henderson NL, Mason RC (2001). Juxtaglomerular cell tumor in pregnancy. Obstet Gynecol.

[5] Kim HJ, Kim CH, Choi YJ, Ayala AG, Amirikachi M, Ro JY (2006). Juxtaglomerular cell tumor of kidney with CD34 and CD117 immunoreactivity: report of 5 cases. Arch Pathol Lab Med.

[6] Lachvac L, Svajdler M, Valansky L, Nagy V, Benicky M, Frohlichova L, Nyitrayova O (2011). Juxtaglomerular cell tumor, causing fetal demise. Int Urol Nephrol.

[7] Shin YS, Cha JS, Kang MJ, Park JK, Kim HJ, Kim MK (2012). Newly developed hypertension due to juxtaglomerular cell tumor in pregnancy. Clin Nephrol.

[8] Ohashi Y, Kobayashi S, Arai T, Nemoto T, Aoki C, Nagata M, Sakai K (2014). Focal segmental glomerulosclerosis secondary to juxtaglomerular cell tumor during pregnancy: a case report. Case Rep Nephrol Urol.

[9] Diker-Cohen T, Abraham SB, Rauschecker M (2014). Reninoma presenting in pregnancy. J Clin Endocrinol Metab.

[10] Xue M, Chen Y, Zhang J, Guan Y, Yang L, Wu B (2017). Reninoma coexisting with adrenal adenoma during pregnancy: a case report. Oncol Lett.

[11] Fu X, Deng G, Wang K, Shao C, Xie LP (2023). Pregnancy complicated by juxtaglomerular cell tumor of the kidney: a case report. World J Clin Cases.

[12] Henderson JT, Vesco KK, Senger CA, Thomas RG, Redmond N (2021). Aspirin use to prevent preeclampsia and related morbidity and mortality: updated evidence report and systematic review for the US Preventive Services Task Force. JAMA.

[13] Gu WJ, Zhang LX, Jin N (2016). Rare and curable renin-mediated hypertension: a series of six cases and a literature review. J Pediatr Endocrinol Metab.

[14] Kuroda N, Gotoda H, Ohe C (2011). Review of juxtaglomerular cell tumor with focus on pathobiological aspect. Diagn Pathol.

[15] Wong L, Hsu TH, Perlroth MG, Hofmann LV, Haynes CM, Katznelson L (2008). Reninoma: case report and literature review. J Hypertens.

[16] Khalid A, Khan BA, Saeed Z, Atique U, Khan MY, -Ul-Haq I (2023). Epithelioid hepatic angiomyolipoma in pregnancy: a case report. Int J Surg Case Rep.

[17] Miyauchi K, Nakai T, Saito S (2021). Renal interstitial fibroblasts coproduce erythropoietin and renin under anaemic conditions. EBioMedicine.

[18] Park SY, Ham WS, Jung HJ, Jeong W, Kim WT, Rha KH (2008). Robot-assisted laparoscopic partial nephrectomy during pregnancy. J Robot Surg.

[19] Ramirez D, Maurice MJ, Seager C, Haber GP (2016). Robotic partial nephrectomy during pregnancy: case report and special considerations. Urology.

[20] Völler M, Mahmud W, Vallo S, Grabbert M, John P, Khoder WY (2021). A 27-year-old primigravida with a right renal cell carcinoma removed at 30 weeks of gestation by robot-assisted retroperitoneoscopic partial nephrectomy. Am J Case Rep.

